# Varying Electronic Configurations in Compressed Atoms: From the Role of the Spatial Extension of Atomic Orbitals to the Change of Electronic Configuration as an Isobaric Transformation

**DOI:** 10.1021/acs.jctc.0c00443

**Published:** 2020-06-18

**Authors:** Roberto Cammi, Martin Rahm, Roald Hoffmann, N. W. Ashcroft

**Affiliations:** † Department of Chemical Science, Life Science and Environmental Sustainability, University of Parma, 43124 Parma, Italy; ‡ Department of Chemistry and Chemical Engineering, Chalmers University of Technology, SE-412 96 Gothenburg, Sweden; § Department of Chemistry and Chemical Biology, Baker Laboratory, Cornell University, Ithaca, New York 14853, United States; ∥ Laboratory of Atomic and Solid State Physics, Cornell University, Ithaca, New York 14853, United States

## Abstract

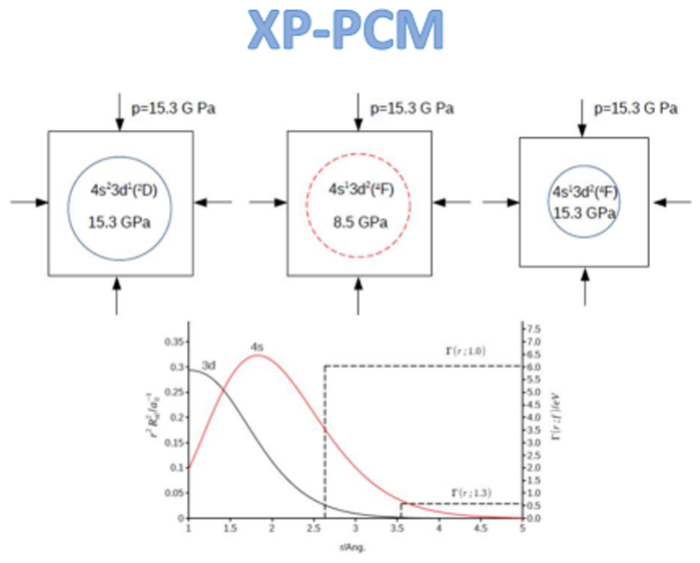

A quantum chemical model for the study of the electronic structure of compressed atoms lends itself to a perturbation-theoretic analysis. It is shown, both analytically and numerically, that the increase of the electronic energy with increasing compression depends on the electronic configuration, as a result of the variable spatial extent of the atomic orbitals involved. The different destabilization of the electronic states may lead to an isobaric change of the ground-state electronic configuration, and the same first-order model paves the way to a simple thermodynamical interpretation of this process.

## 1 Introduction

A characteristic feature of compression, and a source of inherent interest in it, is that the ground-state electronic configuration of an atom (and in atoms in bulk matter, or in compounds) may change as the pressure increases. To put it more dramatically, the Periodic Table changes with pressure. For instance, s-block elements such as K or Cs become transition-metal-like. In their ground states their valence electrons enter preferably 3d orbitals, not 4s. This is not a theoretician’s dream; there is direct experimental information on this, not for atoms, but for the extended elemental solids.

Returning to isolated atoms, this is what we learned in a recent paper^1^ (hereafter called paper I) where we presented a quantum chemical method (the eXtreme Pressure Polarizable Continuum Model, XP-PCM) for the study of the electronic structure in compressed atoms. There, we studied the effects of compression, up to 300 GPa, on the atomic energy levels, configurational energies, and electronegativities of atoms of 93 elements. We found changes in the ground-state electronic configuration of many atoms of the s, d, and f parts of the Periodic Table. In the same paper, we suggested that both the variable destabilization of the electronic configurations and the associated transition pressures can be traced to varying destabilization of the atomic orbitals of the compressed atoms, a consequence of their variable spatial extension. Now we want to shed more light on the nature of this connection.

The objectives of the present paper are 2-fold: (i) to find an explicit functional relationship between the spatial extent of atomic orbitals and the destabilization of the electronic energy of a given electronic configuration under compression and (ii) to analyze in more detail the nontrivial thermodynamic aspects of the isobaric transition between competing electronic configurations. To carry out the requisite analysis, we have considered a version of the XP-PCM method in which the associated quantum chemical problem is set up in the formalism of perturbation theory; the zero-order electronic wave functions of the atoms are expressed as Slater-type atomic orbitals^2^ of the isolated atoms. The utility of this approach will become clear: it gives a functionally and graphically transparent way of understanding what happens.

## 2 XP-PCM Compression of Atomic Systems

In the XP-CM method,^3−8^ a single atom is confined within a spherical cavity inside an external continuum medium transmitting the pressure. The radius, *R*, of the enclosing cavity is related to the van der Waals radius of the free atom *R*_vdW_(9−11) by a scaling factor, *R*(*f*) = *R*_vdW_. An upper value of the scaling factor, *f*_0_ = 1.3, is set as a reference corresponding to low pressure, and lower values of *f* are used to decrease the volume of the cavity and hence increase the compression of the atom.

The medium external to the cavity is characterized by an average electronic charge density, whose magnitude, in turn, depends on the given condition of pressure; that charge density interacts in a repulsive manner with the atom. The pressure acting on the atomic system is computed from the derivative of the electronic energy of the atom, *E*, with respect to the volume, *V*_c_, of the cavity,
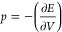
1The electronic energy, *E*, is determined by solving the time-independent Schrödinger equation^a^
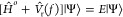
2where Ψ is the wave function of the compressed atom, *H°* the electronic Hamiltonian operator of the isolated atom, and the *V̂*_r_(*f*) operator represents the Pauli repulsion of the atom with the external medium. It is through the Pauli repulsion that the pressure is transmitted to the atomic system. The Pauli repulsion operator *V̂*_r_(*f*) corresponds to a step barrier potential located at the boundary of the atomic cavity, and depends parametrically on the cavity scaling factor *f*. That is,
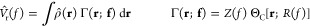
3where *ρ̂*(**r**) = ∫_*i*_^*N*^δ(**r** – **r**_*i*_) is the electron density operator (over the *N* electrons of the molecular system), and Γ(**r**; **f**) is the step barrier potential. The latter potential is made up of Θ_C_[**r**; *R*(*f*)], a spherical Heaviside unit step function of radius *R*(*f*) with the step located at the boundary of the cavity, and *Z*(*f*), the height of the Pauli repulsion barrier. *Z*(*f*) depends on the cavity scaling factor as
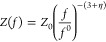
4In eq 4, *Z*_0_ is the step barrier, *f*_0_ is the reference cavity scaling factor, and η is a semiempirical parameter that gauges the strength of the Pauli repulsion. The η Pauli repulsion parameter is determined through comparisons of the computed and experimental pressure–volume equation of state for several substances.^1,8,12^

### 2.1 XP-PCM: A First-Order Perturbation Approximation with Slater Atomic Orbitals

As anticipated in the Introduction, the solution of the XP-PCM electronic problem (2) will be approached within a first-order perturbation theory expansion. In our approach, the confining potential *V̂*_r_(*f*) of eq 3 acts as the perturbation. The zero-order electronic wave functions Ψ^0^ are assumed to be single Slater determinants, composed of Slater-type atomic orbitals^2^ of the free atoms:
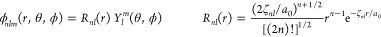
where *ζ*_*nl*_ is the orbital exponent, *a*_0_ is the Bohr radius, *n* is the orbital’s principal quantum number, *n* and *l* are the azimuthal and the magnetic quantum numbers, and *Y*_*l*_^*m*^(θ, φ) is a normalized spherical harmonic function. The zero-order electronic and orbital energies will be taken from density functional theory (DFT) calculations of the unperturbed atom.

Of course, Slater functions, being nodeless polynomials multiplyin exponentials, do not give good approximations of the details (in particular the nodal structure) of orbitals as one approaches the nucleus. But that is not where Pauli repulsion between the atom and the environment occurs (as will be clearly shown in the numerical section); the Pauli repulsion operator is different from zero in the outer regions of the atom. Slater functions can capture well the essential aspect of an exponential falloff of the atomic densities in those outer regions.

Although this first-order perturbation scheme neglects the second-order effects of the compression on the radial distribution function of the atomic orbitals, it is, as we shall see, able to offer physical insight into the effect of pressure on the electronic structure of compressed atoms. The main second-order effect that is omitted in this first-order model is orbital relaxation, which acts to reduce the Pauli repulsion with the surrounding environment.

#### 2.1.1 Orbital Energies, Total Electronic Energy and Pressure: A First-Order Approximation

We start by putting an atom in a given electron configuration into a cavity with variable radius *R*(*f*) and volume *V*_c_(*f*) = 4/3π*R*^3^(*f*). The initial discussion is restricted to what may be loosely called a generalized transition metal element, with a variable occupation of the nd and (*n* + 1)s valence orbitals, i.e., with electronic configurations (*n* + 1)s^*n*^_^s^_*n*d^*n*^_^d^_. The electronic states of a given electronic configuration will be considered within the *L*–*S* Russell–Saunders coupling scheme. It can be shown that, because of the spherical symmetry of the compression operator *V̂*_r_(*f*), the first-order effect on the electronic energy will be the same for all *L*–*S* terms.

The first-order effect of the compression on the electronic energy of the atom is given by the expectation values of the confining operator *V̂*_r_(*f*) in the unperturbed determinantal wave function:
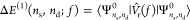
5where Ψ_*n*_s_,*n*_d__^0^ is the zero-order wave function of a state associated with the electronic configuration (*n* + 1)s^*n*^_^s^_*n*d^*n*^_^d^_. The advantage of the Slater function basis is immediately seen, by applying the Slater rules^13^ for the expectation value of a one-body *N*-electron operator, we obtain
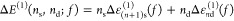
6aand for the first-order total electronic energy
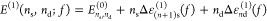
6bwhere *E*_*n*_s_,*n*_d__^(0)^ is the zero-order energy of the given electronic state and Δ*ε*_(*n*+1)s_^(1)^(*f*) and Δ*ε*_*n*d_^(1)^(*f*) are the first-order corrections to the orbital energies. That is,
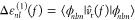
7where the Slater atomic orbitals of the free atom are *φ*_*nlm*_ and *V̂*(*f*) is the one-electron Pauli operator *v̂*(*f*) = δ(*r* – *r*′)*Z*(*f*) Θ_C_(*r*; *R*(*f*)).

From eq 6b, the shift in the electronic energy *E*_*n*_s_,*n*_d__^(1)^ in our atom model depends on first-order corrections to the orbital energies of the nd and (*n* + 1)s valence orbitals and on the occupation number of these orbitals.

In turn, the first-order corrections to the orbital energies Δ*ε*_*nl*_^(1)^(*f*) of eq 7 can be expressed in terms of the spatial extension of the atomic orbital:
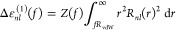
8where *r*^2^*R*_*nl*_(*r*)^2^ is the radial distribution function of the atomic orbital.

The meaning of eq 8 is clear: in our first-order approximation, the increase of the orbital energies is determined by the integrated magnitude of the orbital radial distribution functions in the domain [*fR*_vdW_ – ∞] of the confining potential, times the height, *Z*(*f*), of the confining potential. Hence, *the greater the spatial extent of the radial distribution function in the domain of the confining potential, (r > fR*_*vdW*_*), the greater is the shift of the orbital energy*. Since the (*n* + 1)s orbitals have a greater spatial extension than the nd orbitals,^14^ the shift of the (*n* + 1)s orbital energy will be larger than that of the nd orbitals. This has further implications in our model atom: *the greater the occupation number of the (n + 1)s orbitals, the greater is the shift of the electronic energy*, according to eq 6b.

Another meaningful form of the first-order shift of the electronic energy in a compressed atom follows by substituting eq 8 into eq 6a, to obtain
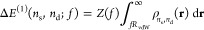
9Here ρ_*n*___s___,*n*___d__(**r**; *n*_s_, *n*_d_) is the total radial density distribution function for the electronic configuration (*n* + 1)s^*n*^_^s^_*n*d^*n*^_^d^_:
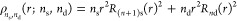
10Note that both eqs 6a and 9 are a direct result of the differential Hellmann–Feynmann theorem^15^ d*E*/dλ = ⟨Ψ|d*Ĥ*/dλ|Ψ⟩/⟨Ψ|Ψ⟩, for the XP-PCM Hamiltonian *Ĥ*(λ) = *Ĥ*° + *λV̂*_r_(*f*).^16^

We complete our XP-PCM first-order theory by determining an analytic form of the pressure experienced by the compressed atom, in a manner analogous to the analytical forms that we have derived above for the orbital energies and for the total electronic energy.

We consider again a model atom in an electronic state with electronic configuration (*n* + 1)s^*n*^_^s^_*n*d^*n*^_^d^_. To obtain an analytic expression for the pressure, we start from the definition given in eq 1 and follow two approaches, formally different but giving the same final analytical expression.

The first approach substitutes the electronic energy *E* with its first-order approximation *E*_*n*_s_,*n*_d__^(1)^ of eq 6b and obtains the pressure by direct differentiation with respect to the cavity volume *V*_c_(*f*):
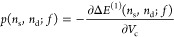
11By applying the differentiation chain rule, we can write
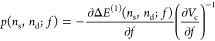
12Here the derivative of the cavity volume with respect to the cavity scaling factor *f* is given by
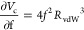
13and, because of the right side of eq 6b, the derivative of the electronic energy can be broken down as a sum of orbital contributions:
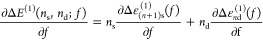
14Then, by introducing eq 8 in eq 12, we obtain the final expression of the pressure as
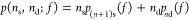
15where *p*(*n* + 1)_s_(*f*), *p*_*n*d_(*f*) are defined by
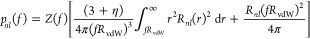
16The quantities *p*_*nl*_(*f*) correspond to the pressure experienced by a single electron occupying the atomic orbital (*nl*) in the compressed atom, and will here be called the “orbital pressure”. We are fully aware that pressure is a macroscopic observable, defined in reality only for an ensemble of atoms or other fundamental units. In fact, we will spend some time later in this paper worrying about just this point: how to relate our “orbital pressure” to a macroscopic ensemble observable.

The above-defined orbital pressure *p*_*nl*_(*f*), in parallel to the shift of the orbital energy Δ*ε*_*nl*_^(1)^, is proportional to the integrated portion of the orbital radial distribution function lying outside of the cavity. Hence, *the further out from the nucleus the orbital’s radial distribution function extends, the higher is the orbital pressure p*_*nl*_. This implies that the electron occupying an (*n* + 1)s orbital experiences a greater orbital pressure than an electron in an *n*d orbital, and that the *greater the occupation number of the (n + 1)s orbitals, the larger is the pressure experienced by the electronic state*. Note that the correlation between the orbital pressure *p*_*nl*_(*f*) and the orbital energy Δ*ε*_*nl*_^(1)^, is made explicit by substituting eq 7 into eq 11. That is,
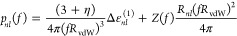
17Hence, the greater is the first-order correction to the orbital energy, the greater is the orbital pressure. Finally, the total pressure ^*n*_s_,*n*_d_^*p*(*f*) can also be expressed in terms of the total radial distribution function ρ(*r*; *n*_s_, *n*_d_) as
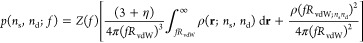
18We next illustrate the second approach for deriving the analytical form of the pressure experienced by our compressed atom. We anticipate that this second method holds also for exact wave function as well as for selected approximate wave function (e.g., Hartree–Fock, DFT, etc.). We begin by exploiting the chain rule of differentiation in eq 1,
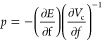
19The Hellmann–Feynman theorem can then be used to evaluate of the derivative of the electronic energy with respect to the cavity scaling factor *f*:^16^
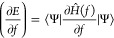
20The electronic Hamiltonian in eq 20 can be considered a parametric function of the cavity scaling factor *f*: *Ĥ*(*f*) = *Ĥ*° + *V̂*_r_(*f*). Hence, eq 20 reduces to the expectation value of the derivative of the XP-PCM Pauli repulsion potential with respect to *f*:
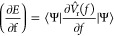
21In turn, by direct differentiation of eqs 3 and 4 we get
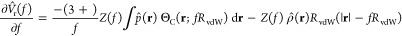
22where δ(|*r*| – *fR*_vdW_ is a Dirac delta function. Substitution of eq 22 into eq 21 gives
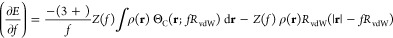
23where ρ(*r*) is the electron density ρ(*r*) = ⟨Ψ|ρ̂(**r**)|Ψ⟩.

Finally, by substituting eqs 19 and 11 into eq 15, we obtain the following analytical expression for the pressure:^16^
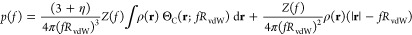
24which, in the case of our first-order approximation, reduces to eq 18.

Before we leave our general considerations, we need to differentiate our approach from that of Daniel Fredrickson and his group,^17^ who have introduced a concept of chemical pressure as a determinant of structure in intermetallics and other inorganic compounds. In the chemical pressure (CP) method, the definition of macroscopic (or internal) pressure *p* is equivalent to the pressure defined with the XP-PCM model (as we can see by comparison of eq 4 of ref (17f) with eq 3 of our paper I). In both the CP and XP-PCM methods the internal pressure is computed as the negative of the derivative of the electronic energy with respect to the volume occupied by the material system (i.e., the unit cell volume in the case of the CP method and the volume of the cavity for XP-PCM method). However, the key (and fruitful) idea of the CP approach is to partition the internal pressure into a pressure distribution (i.e., a scalar field) in the space occupied by the atomic constituents of the compound. This CP distribution is then exploited to analyze the interatomic interactions determining the structure of the material. The XP-PCM decomposition of the pressure through eq 15 has a different aim: analyzing the Pauli repulsive interaction ensuing on compression and its effect on atomic energy levels and configuration energies and, correspondingly, atomic volumes and electronegativities.

## 3 Pressure-Induced Configurational Change in the Scandium Atom

Our XP-PCM first-order perturbation theory allows for a simple qualitative understanding of what happens in the compressed model atom, with variable electronic configuration (*n* + 1)s^*n*^_^s^_*n*d^*n*^_^d^_, in terms of the differences in the spatial extent of the (*n* + 1)s, *n*d orbitals. For a better appreciation of the theory and the understanding it provides, we move a further step, presenting a quantitative implementation.

The numerical application chosen examines the case of the compressed scandium atom in its electronic configuration states 4s^2^3d^1^ (^2^D) and 4s^2^3d^2^ (^4^F). These states are the electronic ground state and the first excited state of the free scandium atom, respectively. As was mentioned in the Introduction, many elements in the s, d part of the Periodic Table show a transition of ground-state electron configuration as a function of pressure (see Figure 12 of paper I), and the scandium atom serves as the prototype for such changes.

### 3.1 Computational Protocol

Early on in the history and lore of using Slater orbitals, it became clear that it is acceptable to use a single Slater function for simulating the distance falloff of the true atomic orbital, but that one needed a so-called double *ζ*_*nl*_ function, effectively a linear combination of two *n*d functions, to capture the shape of an *n*d orbital. Accordingly, in our calculations, the 4s atomic orbital of Sc has been chosen as a single Slater atomic orbital, exponent chosen as by Clementi and Raimondi,^18^ while the 3d atomic orbital has been represented by a linear combination of two Slater orbitals, using the Richardson et al.^19^ fitting. The 4s,3d exponents *ζ*_*nl*_ and the coefficients of the 3d linear combination are reported in Table 1. In the same Table 1 are also reported the zero-order orbital energies *ε*_4s_^0^ and *ε*_3d_^0^ of the 4s and 3d orbitals and the zero-order electronic energies *E*^(0)^(^2^D) and *E*^(0)^(^4^F) for the ^2^D and ^4^F states of free scandium atom. They have been obtained from density functional theory^20,21^ unrestricted calculations using the B3LYP exchange–correlation functional^22^ and the aug-cc-pvtz basis set,^23^ by using the Gaussian 16 suite of programs.^24^

**Table 1:** Exponents (*ζ*_*nl*_) and Coefficients (*c*_*i*_) of the Slater Atomic Orbitals for the 4s and 3d Orbitals Used to Describe the Free Scandium Atom, along with Electronic Energy of the Doublet and Quartet States and the 4s and 3d Orbital Energies at the UB3LYP/cc-pVTZ Level of Theory

ζ_4s_	1.1581
ζ_3d_^1^/ζ_3d_^2^	4.35/1.30
*c*_1_/*c*_2_	0.405688/0.800550
*E*^(0)^(^2^D)	–760.645169*E*_h_
*E*^(0)^(^4^F)	–760.61171*E*_h_
ε_4s_^0^	–4.6450 eV
ε_3d_^0^	–5.0213 eV

For the first-order XP-PCM calculations we have used the same values of the compression cavity parameters, *Z*_0_, η, *f*_0_, *R*_vdW_, which we used in paper I for full-electron XP-PCM/DFT calculations of a compressed scandium atom. That is, *Z*_0_ = 2.968884 × 10^–2^*E*_h_ for the reference value of the confining potential (see eq 3), η = 6 for the Pauli repulsion parameter, *f*_0_= 1.3 for the reference cavity scaling factor, and *R*_vdW_ = 2.63 Å for the van der Waals radius of the scandium atom. The cavity scaling factor of the cavity’s radius *R*(*f*) = *fR*_vdW_ has been varied within the range *f* = 1.3–0.0, with a variable cavity volume *V*_*c*__(*f*)_ = 170–75 Å^3^. This volume difference corresponds to a range of pressure *p* = 0.2–26 GPa for the scandium atom in the electronic state 4s^2^3d^1^ (^2^D). All the first-order XP-PCM numerical calculations have been performed using the Mathematica software package.^25^

### 3.2 Spatial Extent of the 4s and 3d Orbitals

As we showed in the previous Section 2, the effects of compression on a model atom with variable electronic configuration (*n* + 1)s^*n*^_^s^_*n*d^*n*^_^d^_ have their origin in the different spatial extent of the (*n* + 1)s and *n*d orbitals.

**Figure 1:**
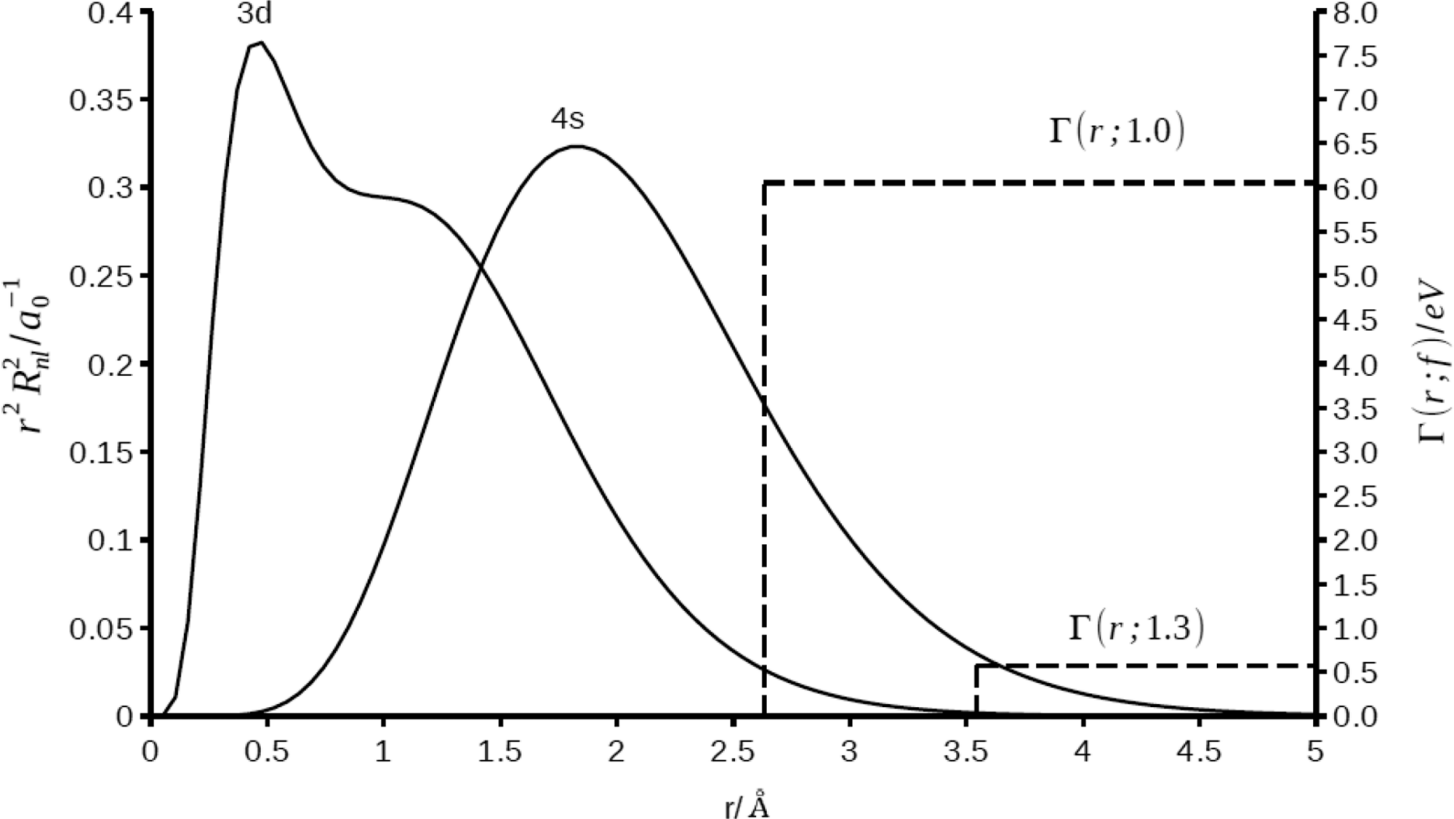
Radial distribution function *r*^2^*R*_*nl*_^2^ (continuous line, left *y*-axis, *a*_o_^–1^) of the 4s and 3d orbitals in the free scandium atom and the XP-PCM confining potential Γ(*r*; *f*) (dashed line, right *y*-axis, eV) for the largest (*f* = 1.3) and the smaller (*f* = 1.0) cavity scaling factors, respectively, for the lower and largest compression of the scandium atom.

In the case of the scandium atom, the relevant comparison is between the 4s and 3d atomic orbitals. In Figure 1 we show the radial distribution functions *r*^2^*R*_*nl*_(*r*)^2^ of these two Slater atomic orbitals of the scandium atom as function of the distance of the electron (*r* = 0–5 Å) from the nucleus. We note that the 3d radial distribution function, with a maximum at *r* = 0.5 Å and a shoulder at *r* = 1.2 Å, reflects the representation of the 3d orbitals as a linear combination of two Slater basis functions, as described previously in the computational protocol. In the same Figure 1 we also show the XP-PCM confining potential, Γ(*r*; *f*) = *Z*(*f*)Θ(*r*; *R*(*f*)), for two different cavity scaling factors (*f* = 1.3 and 1.0). In Table 2 we report the values of the average location, ⟨*r*⟩, of the electron in 4s and 3d orbitals, and the integrated portion (*I*_*nl*_(*f*) = ∫_∞_^*f*_RvdW_^*r*^2^*R*_*nl*_(*r*)^2^ d*r*) of their radial distribution functions penetrating the confining potential Γ(*r*; *f*).

**Table 2:** Spatial Extension of the 4s and 3d Orbitals of the Free Scandium Atom: Mean Distance (Å^3^) of an Electron from the Nucleus, ⟨*r*⟩, and Integrated Portion, *I*_*nl*_(*f*), of the Radial Distribution Function Penetrating the Confining Potential

		*I*_*nl*_(*f*) = ∫_∞_^*f*_RvdW_^*r*^2^*R*_*nl*_(*r*)^2^ d*r*	
atomic orbital	⟨*r*⟩/Å^3^	*f* = 1.3	*f* = 1.0
3d	1.11	0.0015	0.0173
4s	2.04	0.0381	0.1897

With an average location ⟨*r*⟩_3d_ = 1.11 Å, the 3d orbital is closer to the nucleus and further from the outer part of the atoms. The penetration of the 3d orbtial into the confining potential Γ(*r*; *f* = 1.0) integrates to only *I*_3d_(*f*) = 0.017, meaning that an electron occupying this orbital has only a probability of 1.7% to be exposed to the confining potential.

In contrast, the 4s orbital is far from the nucleus. The average electron location ⟨*r*⟩_4s_ = 2.04 Å and the integrated penetration into the confining potential Γ(*r*; *f* = 1.0) is *I*_4s_(*f*) = 0.190. The value of the 4s integrated penetration implies that an electron occupying this orbital has an order of magnitude higher probability than the 3d orbtial to be exposed to the confining potential. Note that this quantitative difference between the spatial extension of the 4s and 3d orbitals only holds in our first-order perturbation scheme, where we rely on the unperturbed 4s and 3d orbitals of the isolated scandium atom (at *p* = 1 atm). The orbitals are effectively frozen; in a more complete theory the 4s and 3d orbitals would change their spatial extension with compression, and would do so to a different degree.

These differences between the spatial extension of the 4s and 3d orbitals have far-reaching consequences on the electronic structure of our simplified model of the compressed scandium atom.

### 3.3 Destabilization of Orbitals and Crossing of the Electronic States upon Shrinking of the Cavity Volume

**Figure 2:**
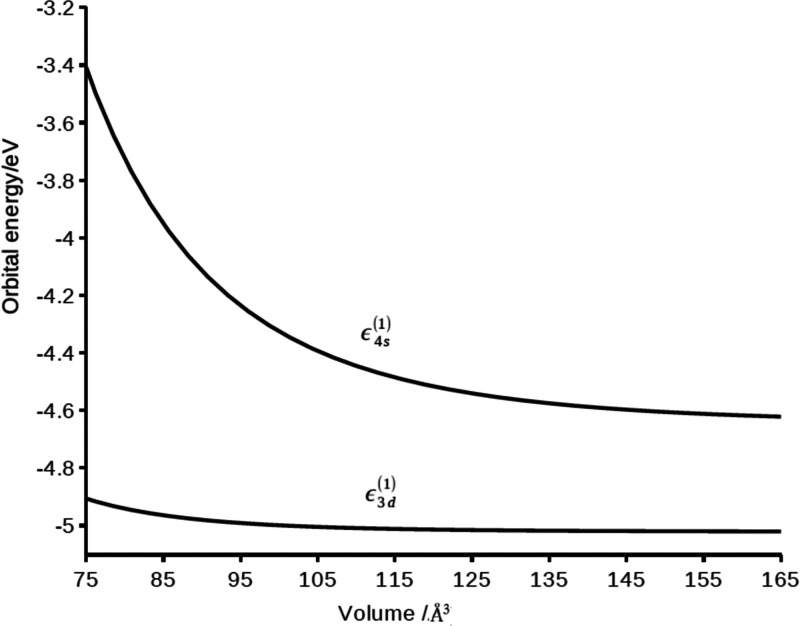
Compressed scandium atom: first-order orbital energies (eV) *ε*_*nl*_^(1)^ of the 4s and 3d orbitals as a function of the compression volume (Å^3^).

Since the 4s orbital of the scandium atom is more exposed to the confining potential than the 3d orbitals, the shift of the 4s orbital energy with compression will be much larger than the shift of the 3d orbital energy. In Figure 2 we show the first-order orbital energies *ε*_4s_^(1)^ and *ε*_3d_^(1)^ as a function of the volume of the compression cavity. The orbital energies *ε*_(nl)_^(1)^ are given by the sum of the zero-order orbital energies *ε*_(nl)_^0^ (given in Table 1) and Δ*ε*_(nl)_^(1)^, the first-order orbital shifts computed according to eq 7: *ε*_(nl)_^(1)^ = *ε*_(nl)_^0^ + *ε*_*nl*_^(1)^.

The orbital energy of the 4s orbital is *ε*_(4s)_^(1)^ = −4.64 eV at the largest cavity volume (*V*_c_ = 160 Å^3^, with *f* = 1.3) and is near the 4s zero-order orbital energy of the free scandium atom. The orbital energy *ε*_4s_^(1)^ increases monotonically with decrease of the cavity volume, up to −3.4 eV at *V*_c_ = 75Å^3^, with a destabilization of 1.2 eV with respect to the orbital energy of the free scandium atom. Over the same range the destabilization of the orbital energy *ε*_3d_^(1)^ of the 3d orbitals is only 0.12 eV, 1 order of magnitude less than the shift of the 4s orbital energy.

Since for Sc the 4s orbital is higher in energy than the 3d orbital (see Table 1), the 4s–3d energy gap in this atom increases with compression. In our first-order model the 4s–3d energy gap is predicted to increase from 0.38 eV at a cavity volume of 160 Å^3^ to 2.72 eV at a cavity volume of 75 Å^3^. The reader will have noticed that if one went by just the orbital energies, one might think Sc would have all of its 3 electrons in 3d orbitals. This, of course, does not happen; one has to account properly for electron interaction in estimating the state energy.^26^

**Figure 3:**
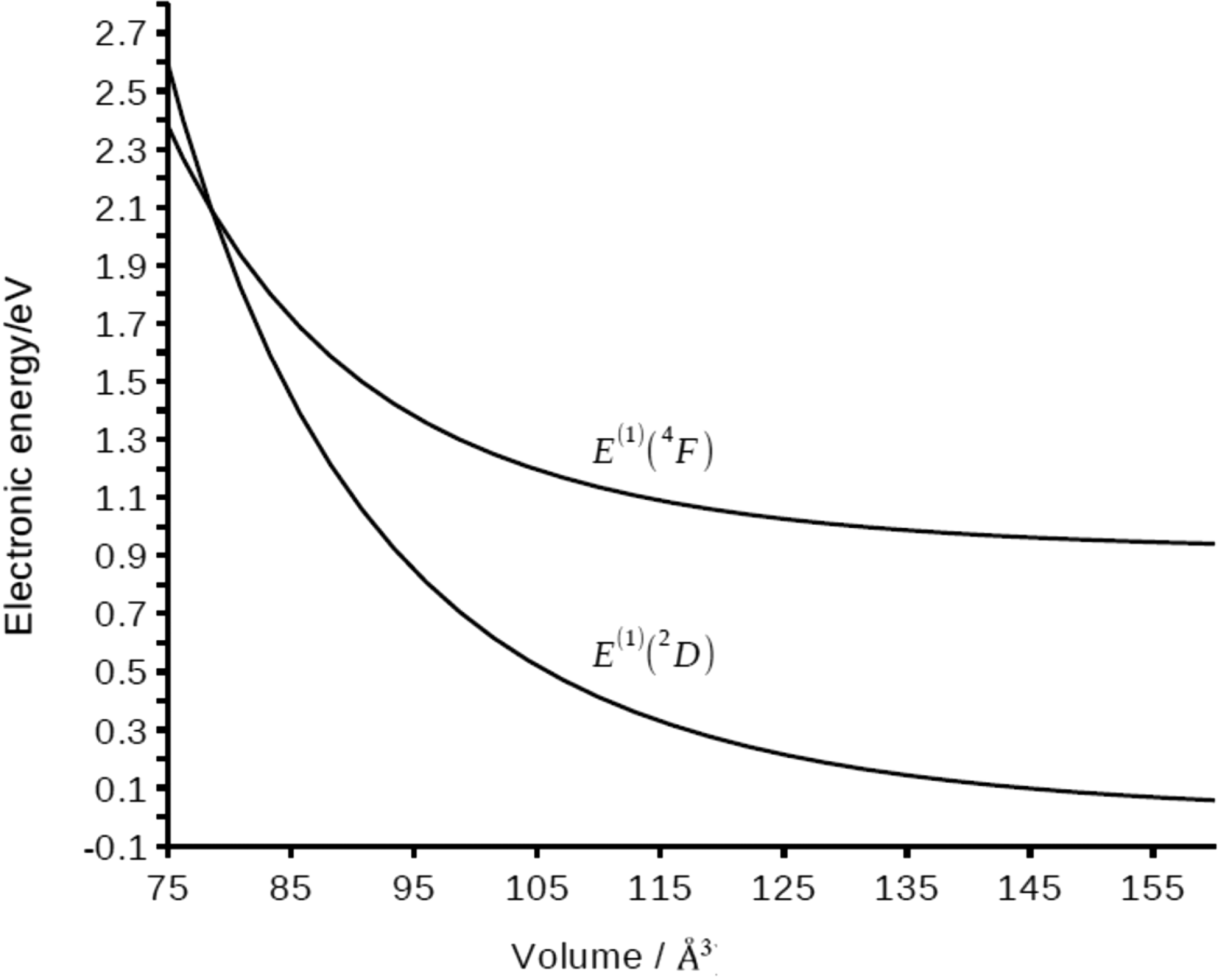
First-order electronic energies (eV) of the 4s^2^3d^1^(^2^D) and 4s^1^3d^2^(^4^F) states of the compressed scandium atom, approximated using eq 7.

In Figure 3 we show the values (eV) of the electronic energies *E*^(1)^(2D) and *E*^(1)^(^4^F) as a function of the volume of the compression cavity. The electronic energies have been computed from eq 6b by introducing the corresponding values of the zero-order electronic energies, the 4s and 3d occupation numbers, and the first-order shifts of the orbital energies Δ*ε*_(4s)_^(1)^, Δ*ε*_(3d)_^(1)^. That is,
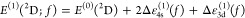

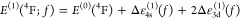


All the values of the electronic energies of Figure 3 are reported relative to the zero-order electronic energy of the ground state *E*^(0)^(^2^D) of the free scandium atom. The ground state *E*^(1)^(^2^D) of the free scandium atom has two electrons in the 4s orbital, and its energy increases with compression, starting from a value near to zero up to 2.6 eV at the cavity volume of 75 Å^3^. In contrast, the electronic energy of the state *E*^(0)^(^4^F), the first excited state (0.95 eV), with only one electron in the 4s orbital, shows a lower increase, of 1.4 eV for the higher compression.

In Table 3 we compare for reference the first-order XP-PCM results of the electronic energies with our previuos full-electron XP-PCM/DFT results of paper I. Its evident that for smaller volumes of the cavity the first-order XP-PCM overestimate the energy destabilization with respect to XP-PCM/DFT. These results are not unexpected, as the first-order method neglects the contraction of the atom 4s and 3d orbitals upon the confining Pauli repulsion potential.

**Table 3:** Comparison between the First-Order XP-PCM and Full-Electron XP-PCM/DFT [1] Results for the Shift of the Electronic Energy Δ*E*^(1)^ (eV), of the Electronic States ^2^D and ^4^F as a Function of the Cavity Volume, *V*_c_

	Δ*E*^(1)^(^2^D)		Δ*E*^(1)^(^4^F)	
*V*_c_/Å^3^	first-order XP-PCM	XP-PCM/DFT	first-order XP-PCM	XP-PCM/DFT
167	0.0	0.0	0.0	0.0
131	0.2	0.2	0.1	0.1
101	0.6	0.4	0.3	0.4
76	2.4	1.4	1.4	1.1

Coming back to the first-order XP-PCM results, we observe a decrease of the ^4^F–^2^D energy gap with decrease of the volume of the cavity. A crossing of the electronic energies at the cavity volume of 80 Å^3^ corresponds to a cavity scaling factor f* = 1.05. For reference, we note that the XP-PCM/DFT results of paper I presented this crossing point at the smaller cavity volume of 60 Å^3^.

The crossing point of the electronic energies is reached when the energy gap of the zero-order electronic energies of the ^4^F and ^2^D state of the free scandium atom is exactly compensated by the difference between the shifts of the 4s,3d orbital in the compressed scandium. That is,
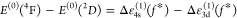
*However, the crossing point of the electronic energies of Figure 3 should not be taken as the point where the ground-state electronic configurations switch*. Transitions between electronic configurations are isobaric processes, in which the pressure experienced by the compressed atom is the same in both of the competing electronic configurations. The crossing point of Figure 3 is not isobaric, instead it corresponds to a transition in which the volume of the compression cavity remains constant. The pressures experienced by the competing electronic configurations in Figure 3 are, according to eq 15, not equal because of their different orbital occupation.

### 3.4 Pressure Dependence of the 4s^2^3d^1^(^4^D),4s^1^3d^2^(^4^F) States of the Scandium Atom

From the XP-PCM first-order theory (eq 15) we can think of a given electronic state as experiencing a certain pressure depending on the associated electronic configuration. This dependence is determined by the orbital occupations and by the pressure experienced by an electron occupying the orbitals, the orbital pressures. In our model atom with variable electronic configuration (*n* + 1)s^*n*^_^s^_*n*d^*n*^_^d^_ the “orbital pressure” of the (*n* + 1)s orbital is greater. Therefore the larger the occupation number of the (*n* + 1)s orbitals the larger is the pressure experienced by an electronic state.

**Figure 4:**
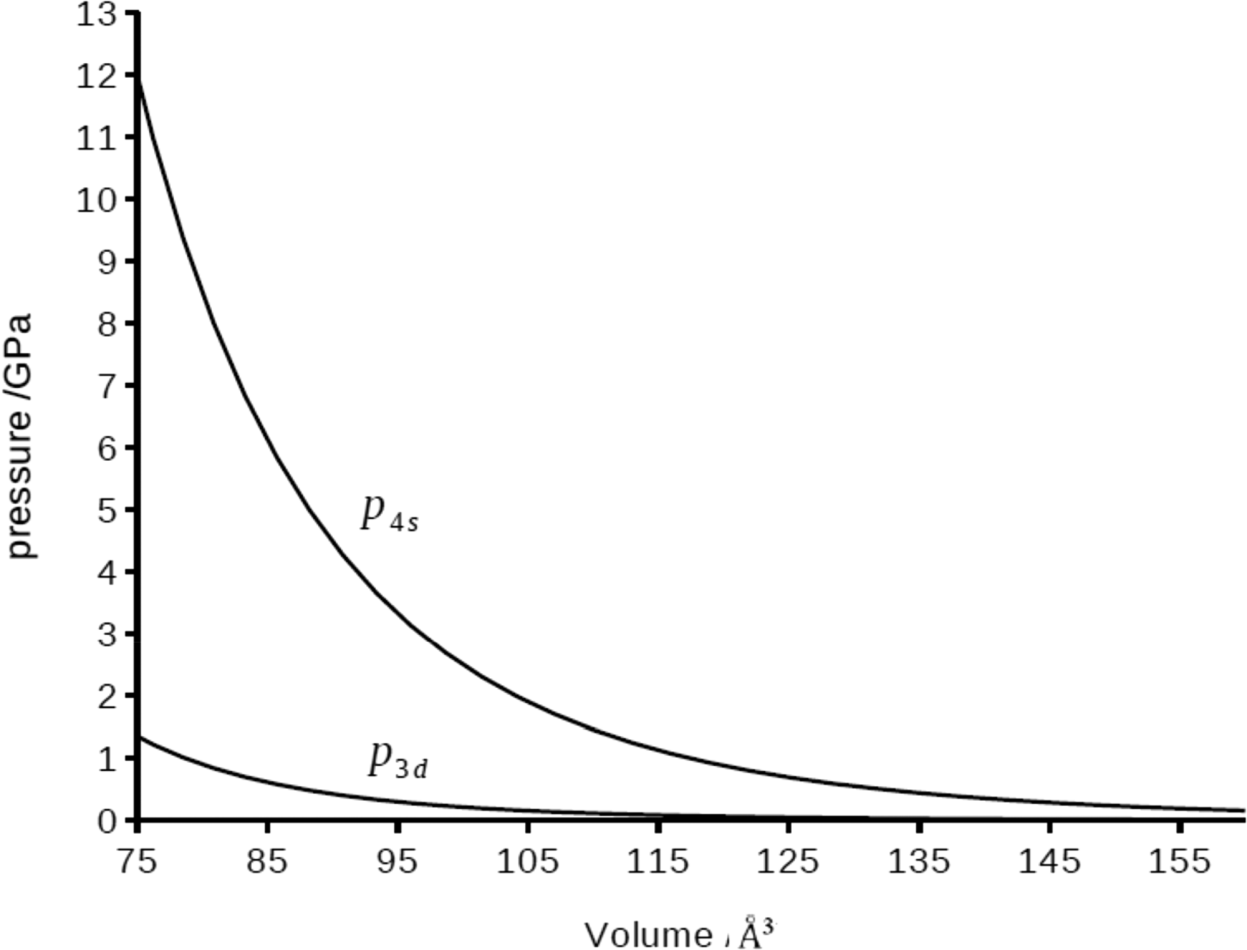
Orbital pressures *p*_*nl*_ (GPa) of the 4s and 3d atomic orbitals in a compressed scandium atom as a function of the compression volume (Å^3^).

In Figure 4 we show the orbital pressures of the Sc 4s and 3d orbitals, which have been computed with eq 14, as a function of the volume of the compression cavity. The orbital pressure experienced by an electron in a 4s orbital is clearly larger (almost by 1 order of magnitude) than that of an electron in a 3d orbital. At higher compression (*V*_c_ = 75 Å^3^), the 4s and 3d orbital pressures are 12 and 1.5 GPa, respectively.

**Figure 5:**
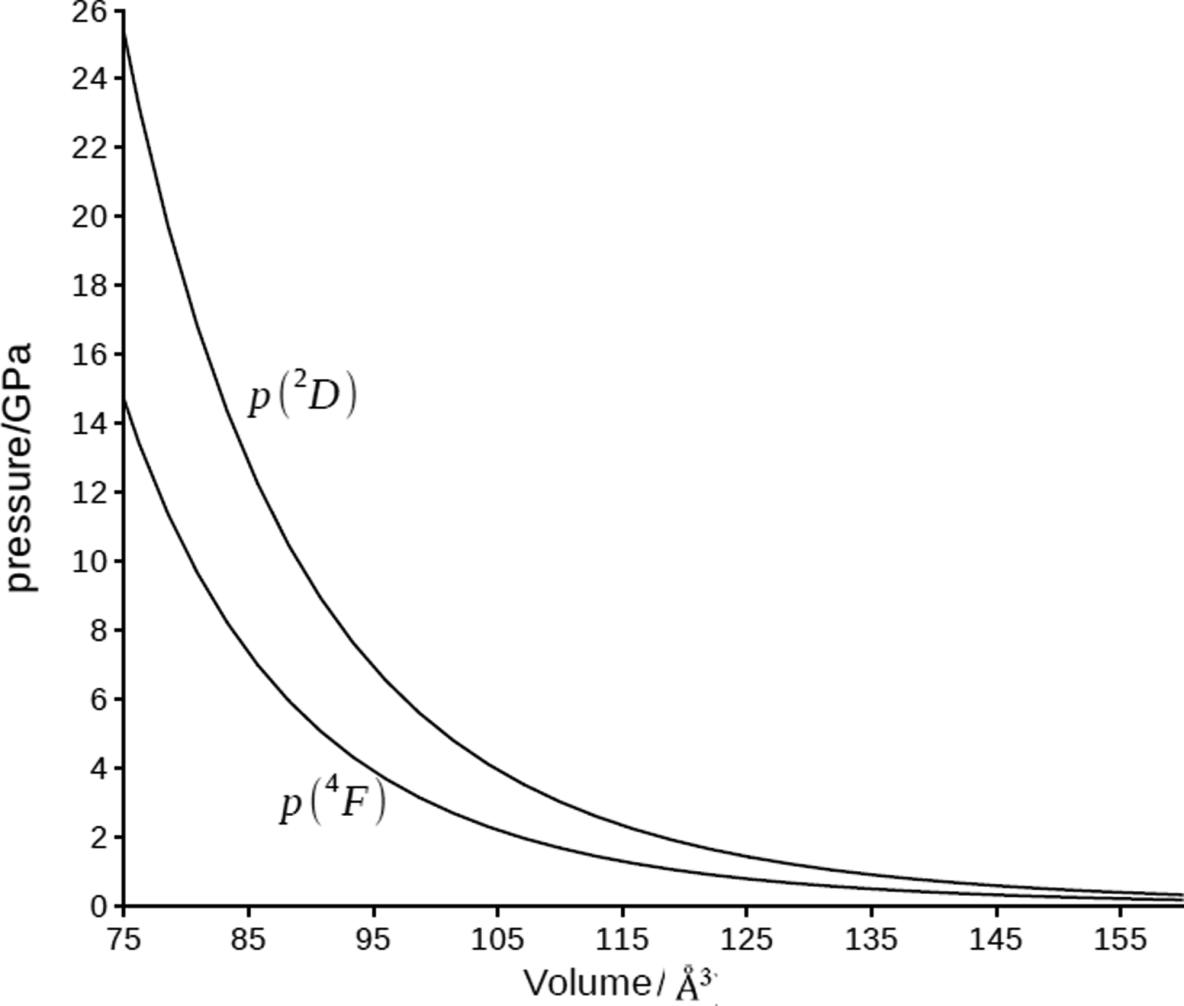
Pressure (in GPa) of the electronic configuration states 4s^2^3d^1^ (^2^D) and 4s^1^3d^2^ (^4^F), in a compressed scandium atom as a function of the compression volume Å^3^.

In Figure 5 we show the pressure acting on the scandium atom in its 4s^2^3d^1^(^2^D) and 4s^1^3d^2^(^4^F)) electronic states, computed from eq 13. As expected, the pressure experienced by the electronic state ^2^D is larger than that of the ^4^F state. In particular, at the cavity volume (*V*_c_ = 75 Å^3^, Figure 3) corresponding to the crossing of the electronic energies, the ^2^D and ^4^F states experience the pressures of 19 and 11 GPa, respectively.

We close this subsection by comparing in Table 4 the first-order XP-PCM results of the pressure acting on the electronic states ^2^D, ^4^F the with those obtained in our previous paper I with the full-electron XP-PCM/DFT calculations. The agreement between the two methods is reasonable, especially when considering lower degrees of compression. A general overestimation of pressures using the first-order XP-PCM is expected as the 4s and 3d orbitals are not allowed to contract.

**Table 4:** Comparison between the the First-Order XP-PCM and Full-Electron XP-PCM/DFT^1^ Results for the Pressure, *p* (GPa), Experienced by the Electronic States ^2^D and ^4^F as a Function of the Cavity Volume, *V*_c_ (Å^3^)

	*p*(^2^D)/GPa		*p*(^4^F)/GPa	
*V*_c_/Å^3^	first XP-PCM	XP-PCM/DFT	first XP-PCM	XP-PCM/DFT
167	0.2	0.3	0.1	0.2
131	1.0	0.9	0.6	0.8
101	4.8	3.2	2.7	2.6
76	23.2	10.7	8.4	13.4

### 3.5 Viewing Electronic Transitions as Isobaric Processes

**Figure 6:**
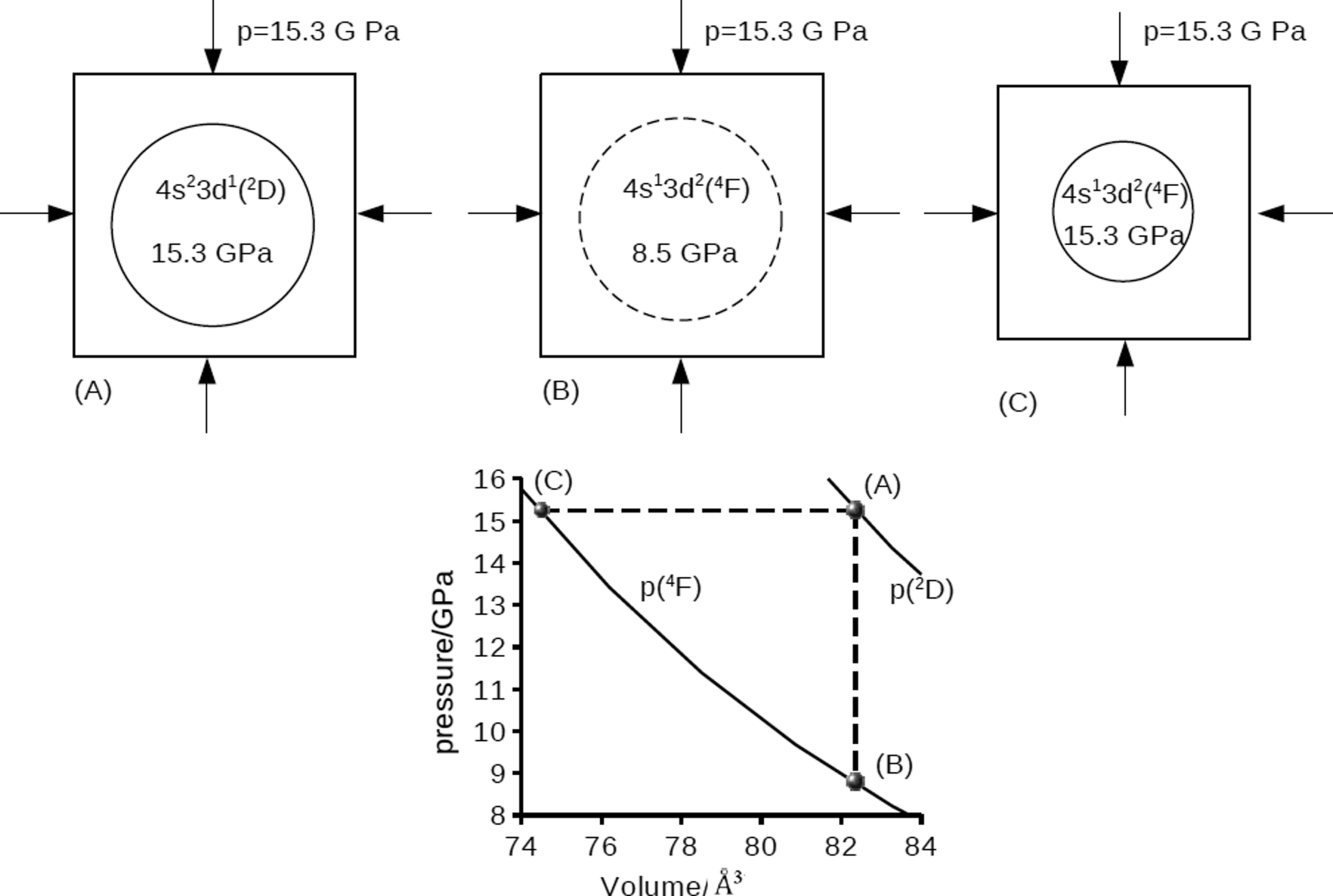
Schematic representation of the isobaric (*p* = 15.3 GPa) electron configurational transition, 4s^2^3d^1^(2D) → 4s^1^3d^2^(^4^F) , in a statistical ensemble of compressed scandium atoms.

Let us consider explicitly how a microscopic electronic configuration transition of a single atom can be connected to a macroscopic isobaric process. To establish this connection, we introduce a statistical ensemble^27^ in which each element is a single compressed atom connected to an external medium transmitting the pressure (see Figure 6). The ensemble is assumed to be at 0 K.

An isobaric change of the electronic configuration of the ensemble can be described as follows: First we consider an initial state of the ensemble where all the scandium atoms are in the electronic configuration state 4s^2^3d^1^(^2^D). This initial state, with its associated volume and pressure, is denoted as (A) in Figure 6. For the model Sc atom, at a pressure of 15.3 GPa the atom has a computed volume of 83.4 Å^3^. The pressure experienced locally by the scandium atom is equal to the macroscopic pressure applied to the ensemble.

In a second step we switch the electronic state of each atom from 4s^2^3d^1^(^2^D) to 4s^1^3d^2^(^4^F) while maintaining a constant volume. According to Figure 5, the pressure experienced locally by the scandium atom drops to *p* = 8.5 GPa with this transition. The resulting state of the system, denoted as (B) in Figure 6, is no longer in thermodynamic equilibrium since the local pressure is lower than the macroscopic pressure (*p* = 15.3 GPa) applied to the ensemble.

In the third step the system is allowed to equilibrate by the increasing the pressure expereinced by the scandium atom in its electronic state 4s^2^3d^1^(^4^F). The final state, denoted as (C) in Figure 6, corresponds to a cavity volume of 74.5 Å^3^, and a pressure of 15.3 GPa.

The total transformation from the initial state A to the final state C corresponds to an isobaric process. The associated change in enthalpy for this process is given by
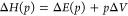
25where Δ*E*(*p*) is the variation of the internal electronic energy,
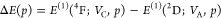
26*p* is the transition pressure, and Δ*V* = *V*_C_ – *V*_A_ is the variation of the cavity volume.

**Figure 7:**
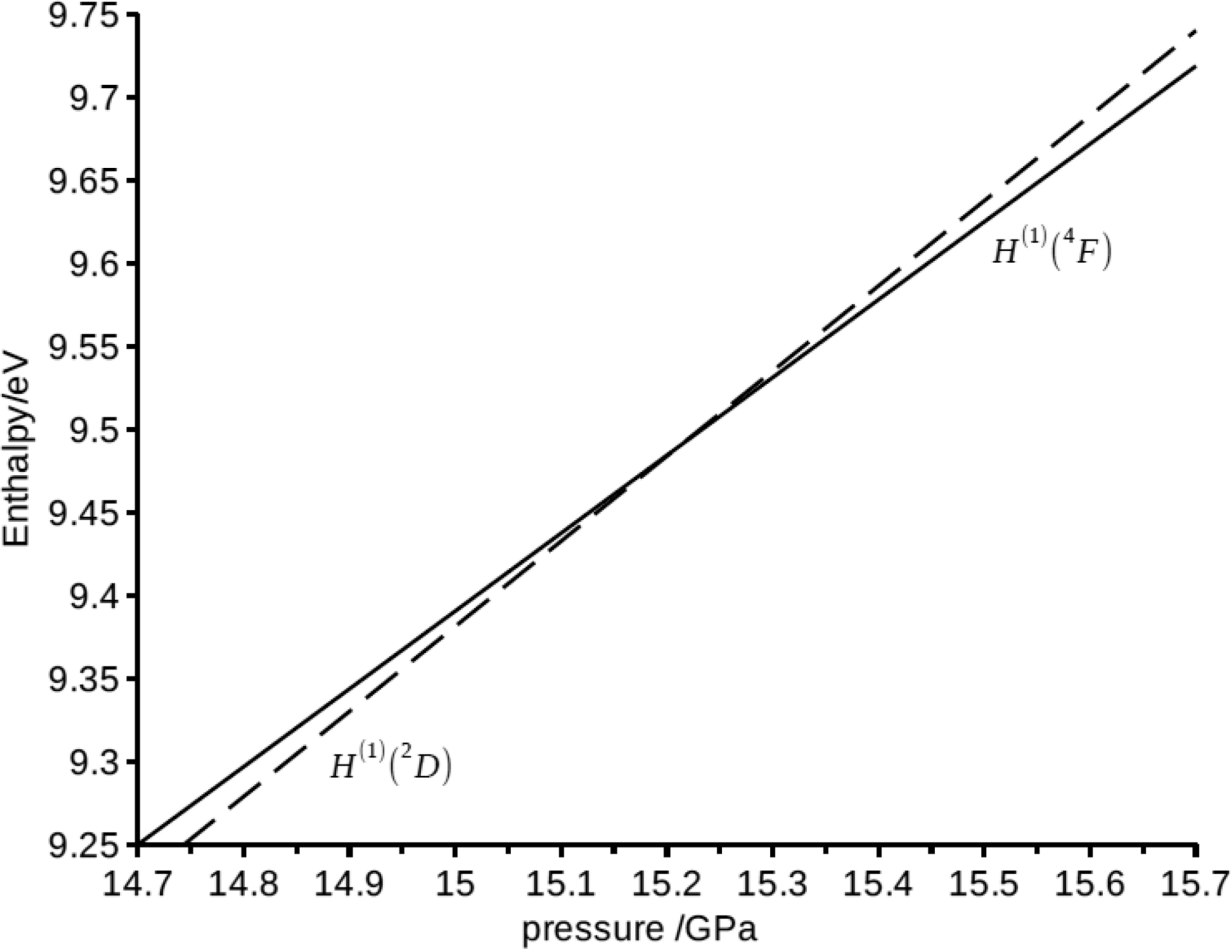
Enthalpy, *H* (eV), of the electronic configuration states 4s^2^3d^1^(^2^D) (dashed line) and 4s^2^3d^1^(^2^*F*) (continuous line) as a function of the pressure *p* (GPa). The crossing point at *p* = 15.3 GPa corresponds to the isobaric change of the ground state of the electronic configuration 4s^2^3d^1^(^2^D) → 4s^2^3d^2^(^4^F).

Because we are considering an ensemble at *T* = 0 K, the isobaric transition of the electronic configuration must occur at a pressure such that the variation of the enthalpy is zero, Δ*H*(*p*) = 0; i.e., the process is iso-enthalpic. In Figure 7 we show the enthalpies as of the electronic configurations 4s^2^3d^1^(^2^D) and 4s^1^3d^2^(^4^F) as a function of pressure. The crossing point of the enthalpies at 15.3 GPa corresponds to the isobaric ground-state transformation of the compressed scandium atom. For reference, we note that the predicted transition pressure from the all-electron DFT-based XP-PCM calculations in paper I was 14 GPa. This fortuitous correspondence is most likely due a error cancellation of the first-order XP-PCM method.

The isobaric process of electronic configuration switching that we have analyzed has a simple thermodynamic interpretation. Since the process is isoenthalpic Δ*H*(*p*) = 0, from eqs 25 and 26, it must hold that
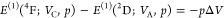
27That is, the variation of the internal energy due to the change of electronic configuration in this isobaric transformation is supported by the mechanical work, −*p*Δ*V*, exerted by the external pressure on the atom. Note that at a temperature different from 0 K, a minor additional role can be played by the electronic entropy, due to the variation of the electronic degeneracy of the scandium atom upon compression.

## 4 Conclusions

This work has been motivated by the desire to find an explicit functional connection between the spatial extent of atomic orbitals and the destabilization of the electronic energy in compressed atoms. To this end, we have presented a first-order perturbation theory of the XP-PCM model, the same quantum chemical model that we have previously used in its all-electron DFT version for the study of the compressed atoms of 96 elements of the periodic table.

In the XP-PCM model, the pressure is generated by a step spherical confining potential of variable size for the electrons of the atom. The first-order perturbation analysis shows that the destabilization of the electronic energy is given by the sum of contributions from the occupied orbitals. And in turn, each orbital destabilization depends on the spatial extension of the atomic orbital, which determines the exposition of electrons potentially occupying such orbitals to the spherical confining potential. The rule that emerges, not surprising, is that the greater the spatial extension, the larger is the orbital destabilization.

A typical (and important) case of the compressed scandium atom illustrates the theory in numerical detail. The different spatial extension of 4s and 3d orbitals is at the origin of the different destabilization of the ^2^D(4s^2^3d^1^) and ^4^F(4s^1^3d^2^) electronic states in the scandium atom, which in turn leads to a switch of the ground-state electronic configuration of this system. The same first-order XP-PCM theory has also been instrumental in a careful thermodynamic analysis of changes to the ground-state electronic configuration as isobaric transitions. The energetic cost of the switching of the ground-state electronic configuration is supplied by the mechanical work exerted by the medium transmitting the pressure.

The scandium atom under pressure has served as a clear pedagogical example of the behavior of its electronic configurations, traced in turn to the different spatial extension of its 4s and 3d orbitals. However, the first-order XP-PCM theory applies to any generic transition metal atoms, both of the first row and of the second and third rows, where the different spatial extension is between 5s/4d and 6s/5d atomic orbitals, respectively. By exploiting the pertinent Slater atomic orbitals parameters proposed by Richardson et al.,^19^ by Clementi et al.,^18,29^ and by Gray and Basch,^30^ specific numerical applications may be obtained.

The first-order XP-PCM model, which permits an analytical solution, may become a useful simple guide^28^ in the understanding of the electronic structure of compressed atoms. However, if we should want more detailed functional connections between the shape and extent of the atomic orbitals and the destabilization of the electronic energy of compressed atoms, further analysis is needed. Indeed, a limitation of the first-order theory XP-PCM is the neglect of the contraction of the atomic orbitals occasioned by the confining potential. Such contraction may modify, to a variable extent, the relative spatial extension of the atomic orbitals. We may anticipate an important role here to be played by the orthonormality constraint between the atomic orbitals. A next step in the analysis of the electronic structure of compressed atoms will be the explicit calculation of the compressibility of atomic orbitals.

## Footnotes

a For sake of simplicity, we have neglected the electrostatic interaction with the external medium, which is considered in the standard XP-PCM method.^3^
